# Titin‐Mimetic Phase Separation Enables a High‐Performance Dielectric Elastomer for Artificial Muscles

**DOI:** 10.1002/advs.78715

**Published:** 2026-11-09

**Authors:** Chengzhen Du, Lijuan Yin, Yujie Chen, Xichen Yang, Zhi‐Min Dang, Zimo Pang, Sijia Li, Qunfu Fan, Hezhou Liu

**Affiliations:** ^1^ School of Materials Science and Engineering State Key Laboratory of Metal Matrix Composites Shanghai Jiao Tong University Shanghai China; ^2^ State Key Laboratory of Smart Power Distribution Equipment and System Hebei University of Technology Tianjin China; ^3^ National Engineering Research Center of Special Equipment and Power System for Ship and Marine Engineering Shanghai China; ^4^ State Key Laboratory of Power System Operation and Control Department of Electrical Engineering Tsinghua University Beijing China

**Keywords:** artificial muscle, dielectric elastomer, phase separation, self‐healing, strain‐stiffening

## Abstract

Dielectric elastomers (DEs) are promising candidates for soft robots to achieve efficient electromechanical conversion and lightweight integration. However, conventional homogeneous DE networks face multiple inherent contradictions among compliance, robustness, and dielectric performance due to the lack of differentiated structural design at the molecular scale. Inspired by the hierarchical responsiveness of titin, we present a phase‐separated fluorinated polyacrylate DE that achieves synergistic optimization of electrical and mechanical properties while exhibiting excellent self‐healing capability. The proposed elastomer achieves a maximum actuation strain of 235% and an ultrahigh specific energy density of 386 J kg^−1^ approximately 9.65 times that of natural muscle. As such, our work demonstrates a remarkably comprehensive performance among contemporary advancements. We further demonstrate a biomimetic swimming robot that achieves efficient aquatic locomotion, highlighting the potential of our DE as a suitable candidate for high‐performance artificial muscle.

## Graphical abstract

Inspired by the hierarchical mechanoresponsive structure of titin, a phase‐separated fluorinated polyacrylate dielectric elastomer exhibits large actuation strain (235%), ultrahigh energy density (386 J kg^−1^), and excellent self‐healing capability. A biomimetic swimming robot driven by this elastomer demonstrates efficient aquatic locomotion, highlighting its potential as a high‐performance artificial muscle for soft robotics.

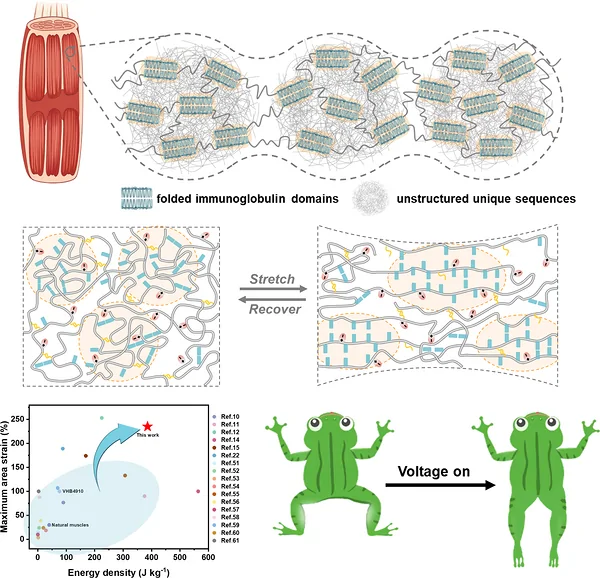

## 1 Introduction

Soft robotics offers significant benefits in terms of safety and adaptability, making it highly promising for medicine, rescue operations, and perception technologies [1, 2, 3, 4, 5]. The expansion of application scenarios and increased functional integration have placed greater demands on the performance of core actuation units [6, 7, 8, 9]. Developing dielectric elastomer actuators (DEAs) with high electromechanical conversion efficiency and robust cyclic stability is a core challenge for advancing soft robotics toward practical applications [10, 11, 12]. Despite remarkable advancements made in high‐performance dielectric elastomers (DEs) in recent years [13, 14, 15, 16], the concurrent realization of large actuation strain, high energy density, and self‐healing capability against mechanical or electrical damage during prolonged service remains elusive.

This dilemma stems from the lack of differentiated structural design at the molecular scale in conventional homogeneous DE networks [17, 18, 19, 20], which engenders the following trade‐offs. For instance, reducing the Young's modulus can significantly improve actuation performance, but it also precipitates electromechanical instability (EMI) [21, 22]. In addition, excessively soft elastomers inevitably suffer from insufficient mechanical strength, leading to fatigue or even fracture, thereby slowing down the response speed [23]. Moreover, approaches such as introducing polar groups or incorporating high‐dielectric‐constant ceramic fillers can enhance the electric field response, but often at the cost of decreased dielectric strength and higher stiffness [24, 25, 26]. Furthermore, homogeneous DEs lack multiphase interfaces to disperse local electric fields and dissipate energy at crack tips, rendering microscopic defects prone to evolving into catastrophic breakdown and rapid crack propagation [27, 28], which inevitably degrades performance and shortens lifetime.

The hierarchical structure of titin offers a paradigm for tackling the aforementioned challenges. As shown in Figure 1a, this structure enables skeletal muscle to withstand large deformations while retaining outstanding toughness, rapid response, and self‐healing properties [29]. Specifically, titin comprises a string of folded immunoglobulin domains and unique unstructured sequences, which serve as entropic springs with different bending rigidities (Figure 1b) [30, 31]. During elongation, the unstructured sequences and folded immunoglobulin domains extend sequentially along the direction of stretch. As the sarcomere length approaches its physiological limit, a small number of folded domains undergo unfolding, which further extends the length of titin and dissipates energy, effectively preventing damage from overstretching [32, 33]. The resulting strain‐stiffening behavior is critical for achieving the exceptional mechanical performance of muscle under dynamic loading conditions. Notably, the reorganization of non‐covalent bonds and the refolding of proteins endow muscles with self‐healing capability.

**FIGURE 1:**
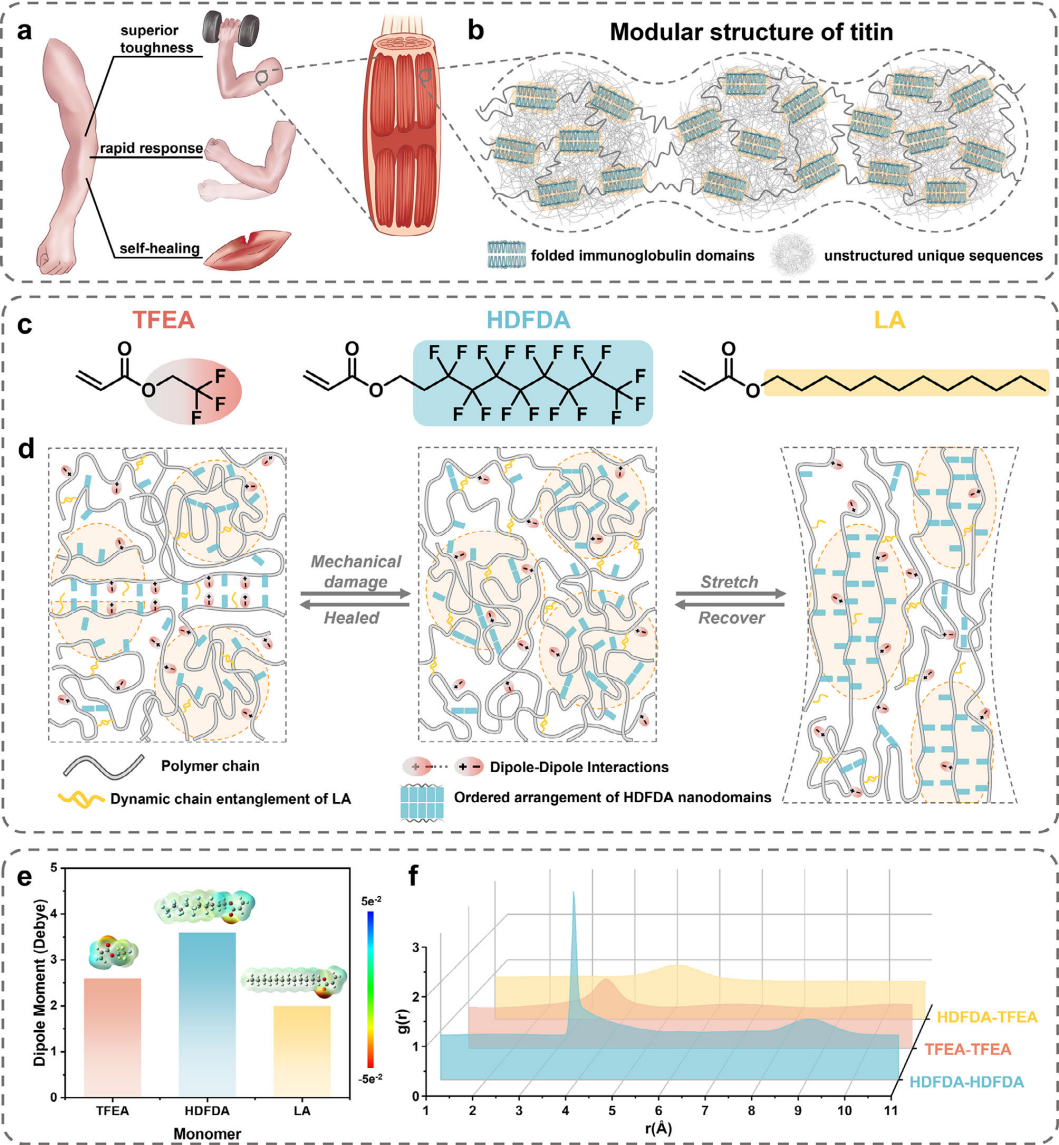
The structure and design concept of titin‐inspired DE. (a) Schematic diagram of human muscle with the capabilities of toughness, rapid responsiveness, and self‐healing. (b) Schematic diagram of the tandem modular construction of titin. (c) Chemical structures of materials used for the synthesis of our high‐performance DE. (d) Phase‐separated structure in the fluorinated DE mimics titin's hierarchical mechanical response. Upon stretching, rigid domains undergo cooperative orientation; LA does not form independent domains but only provides dynamic entanglement. Multiple dynamic physical interactions endow the DE with excellent room‐temperature self‐healing capability. (e) Dipole moments of the monomer. (f) RDFs of different chain segments.

Herein, inspired by titin, we develop a phase‐separated fluorinated polyacrylate DE, in which the hard phase mimics the folded domains to provide mechanical reinforcement, while the soft phase mimics the randomly entangled network to provide softness and elasticity. During deformation, the cooperative orientation of rigid domains generates a hierarchical mechanical response and a strain‐stiffening effect analogous to that observed in titin, which effectively enhances the toughness of the DE and suppresses EMI [34, 35]. Moreover, the synergy of multiple dynamic physical interactions collectively confers rapid response and superior self‐healing capability on the DE. The developed DE attains a maximum actuation strain of 235% and an ultrahigh energy density of 386 J kg^−1^—approximately 9.65 times that of natural muscle, representing a remarkably excellent DE among contemporary advancements. In addition, our DE operates stably above a high energy density exceeding 250 J kg^−1^ for 10 000 cycles. Finally, inspired by purely aquatic frogs, we fabricate a biomimetic swimming robot, demonstrating the substantial potential of this high‐performance artificial muscle.

## 2 Results and Discussion

### 2.1 Polymer Design and Characterization

The titin‐mimetic phase‐separated DE was synthesized via one‐step copolymerization of 2,2,2‐trifluoroethyl acrylate (TFEA), 1H,1H,2H,2H‐heptadecafluorodecyl acrylate (HDFDA), and lauryl acrylate (LA) (Figure 1c). The sample nomenclature and detailed recipes are listed in Table S1. Taking THL30 as an example, in the Fourier‐transform infrared (FTIR) spectrum, the characteristic ν(C = C) peak of the comonomers at 1637 cm^−1^ disappeared, indicating that the comonomer conversion rate approached 100% (Figure S1). The results of ^1^H nuclear magnetic resonance (Figure S2), ^13^C NMR (Figure S3), and ^19^F NMR (Figure S4) confirmed the chemical structure of THL30. Additionally, the weight‐average molecular weight (M_w_) of THL30 was 135 464 Da (Figure S5).

In this strategy, the soft phase TFEA, characterized by a low glass transition temperature, imparts high segmental mobility to the polymer chains, ensuring a low Young's modulus and exceptional flexibility. Simultaneously, the abundance of highly polar CF_3_ groups within TFEA, combined with its favorable chain dynamics, contributes to an outstanding dielectric constant. For the hard phase HDFDA, the pronounced chemical incompatibility between long fluorinated pendant groups and hydrocarbon backbone drives the bulky rigid‐rod‐like side chains to self‐organize into highly ordered nanoscale rigid domains [36, 37]. These nanodomains not only serve as efficient physical crosslinking points to markedly enhance both tensile strength and toughness, but also cooperatively align along the stretching direction under large deformations. Crucially, the entangled network formed by the long alkyl side chains of LA effectively mitigates strain softening and endows the DE with excellent elastic recovery (Figure S6), while LA does not form independent phase‐separated domains (Figure S7). Besides, the interchain diffusion and multiple dynamic physical interactions provide self‐healing potential (Figure 1d).

To gain a deeper understanding of the design rationale, we performed density functional theory (DFT) calculations and molecular dynamics (MD) simulations. According to density functional theory (DFT) calculations, both TFEA (2.59 Debye) and HDFDA (3.59 Debye) exhibited notably high dipole moments (Figure 1e), which were primarily attributed to the strongly polar C‐F bonds. Such high dipole moments facilitated an elevation of the dielectric constant, thereby enhancing the polarization response capability of the DE under an applied electric field. Furthermore, MD simulations were conducted to calculate the radial distribution functions (RDFs) among different chain segments, providing insight into the phase‐separation behavior. As shown in Figure 1f, the RDF for HDFDA‐HDFDA exhibited a sharp first peak at approximately 4.57 Å, indicating strong interactions and an ordered arrangement among the fluorinated side chains. In contrast, the RDFs for TFEA‐TFEA and TFEA‐HDFDA featured broad peaks with significantly reduced intensities, reflecting weak interactions between the soft and hard phases and a disordered distribution within the soft phase. These simulation results provided molecular‐level evidence supporting the self‐assembly behavior of HDFDA.

### 2.2 Composition‐Dependent Evolution of Structure and Properties

Based on the simulation predictions above, we further directly observed the phase‐separated structure using an atomic force microscope (AFM). Figure 2a showed that the HDFDA‐enriched rigid domains (light regions) were homogeneously distributed within the continuous soft phase (dark regions). For THL30, the feature size of the phase‐separated structure was approximately 80 nm. As the HDFDA content gradually increased, enhanced fluorine‐fluorine interactions drove the continued growth of the rigid domains. Consistent with the AFM findings, excellent transparency in the visible range (transmittance ∼90%) was observed for DEs with HDFDA contents less than 50 mol% (Figure 2b). By further increasing HDFDA contents, the size of rigid domains approached the wavelength of visible light, inducing significant light scattering, thus making the DEs noticeably opaque. This was also confirmed by polarized optical microscopy (POM), in which pronounced birefringence appeared at higher HDFDA contents (Figure S8).

**FIGURE 2:**
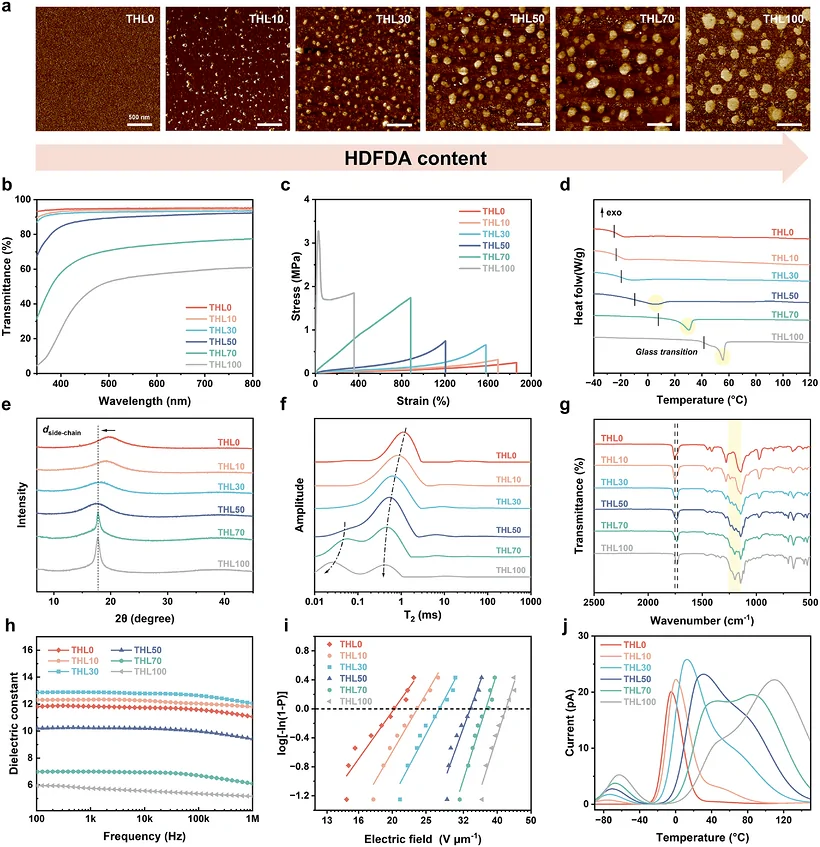
Properties of THL elastomers evolve with HDFDA content. (a) Structural evolution of phase separation of a series of THL elastomers. (b) Visible transmittance spectra of THL elastomers. (c) Stress‐strain curves of THL elastomers. (d) DSC curves with labelled glass transitions. (e) XRD profiles of THL elastomers. The characteristic peaks for the side chains are labelled. (f) Low‐field ^19^F NMR spectra of THL elastomers. (g) FTIR spectra of THL elastomers. (h) Dielectric constants of THL elastomers from 10^2^ to 10^6^ Hz. (i) Weibull distribution of electrical breakdown strength (*n* = 10). (j) TSDC curves of THL elastomers.

HDFDA content also significantly influenced the mechanical properties of the DEs. The calculated Young's modulus from tensile stress‐strain curves increased with rising HDFDA contents (Figure 2c and Figure S9). This trend corroborated the incrementally enhanced interchain associations derived from HDFDA, leading to the formation of a denser rigid physical crosslinking network. Notably, THL30 stood out for its superior mechanical performance, characterized by a low modulus (0.08 MPa), ultrahigh stretchability (a maximum elongation of 1580%), and a prominent strain‐stiffening effect. The strain‐stiffening effect originated from the cooperative rotation and ordered alignment of the initially randomly distributed rigid nanodomains. The process required overcoming strong fluorine‐fluorine interactions and interfacial resistance, thereby manifesting macroscopically as enhanced strength and toughness [38]. When the HDFDA content exceeded 70%, the dense rigid nanodomains severely restricted chain segment mobility, causing a sharp increase in modulus and a substantial decrease in elongation at break. As a result, only THL0, THL10, THL30, and THL50 meet the flexibility and deformability requirements for DEs (Figure S10).

Differential scanning calorimetry (DSC) was performed to elucidate the microstructural evolution induced by changes in HDFDA content. As the HDFDA content increased, the glass transition temperature (*T*_g_) exhibited a gradual upward trend (Figure 2d). The single *T*_g_ observed for all DEs indicated random copolymerization of TFEA and HDFDA with similar reactivities. Moreover, for HDFDA contents less than 50 mol%, the *T*_g_ of DEs remained below room temperature (20°C), demonstrating superior chain flexibility. In contrast, the emergence of distinct melting peaks of DEs with HDFDA contents above 50 mol% confirmed the formation of a smectic phase via HDFDA self‐assembly. X‐ray diffraction (XRD) pattern also corroborated such an ordered structure within the rigid domains. As shown in Figure 2e, the diffraction peak corresponded to the average distance among fluorinated side chains (*d*_side‐chain_). For THL100, the *d*_side‐chain_ of 0.51 nm calculated from the sharp peak was characteristic of the hexagonal in‐plane lattice constant of the smectic phase [39]. The *d*_side‐chain_ exhibited a substantial increase from 0.44 nm at THL0 to 0.51 nm at THL50, after which it reached a plateau at higher HDFDA contents, suggesting close packing among HDFDA moieties (Figure S11). A comparable trend was discernible in the spin‐spin relaxation time (*T*_2_) changes from low‐field ^19^F NMR spectra. As shown in Figure 2f, the main *T*_2_ peak shifted leftward and was accompanied by a decline in intensity, indicating that the HDFDA domains led to reduced mobility of surrounding chain segments (Figure S12). A small *T*_2_ peak emerged on the left side, indicating severely restricted mobility of the fluorinated side chains, which corresponded to the crystallization of the HDFDA moieties.

Fourier transform infrared (FTIR) spectroscopy was additionally used to investigate the internal interactions of THL elastomers. In the C═O stretching vibration region, THL0 exhibited a strong peak at 1755 cm^−1^, accompanied by a weaker shoulder peak at 1725 cm^−1^ (Figure 2g). As the HDFDA content increased, the ordered C═O groups gradually rose, leading to a redshift to 1725 cm^−1^. As revealed by second‐derivative spectra, the CF_2_ stretching vibration peak progressively red‐shifted with gradually increasing intensity (Figure S13). This unambiguously indicated the gradually enhanced HDFDA assemblies with stronger dipole‐dipole interactions.

Low viscoelasticity is essential for enhancing the response speed and actuation bandwidth of DEAs [40, 41]. Due to the rigid domains and the entanglement network suppressing irreversible chain slippage, THL30 showed excellent elasticity and rapid recovery capability. Over ten consecutive cycles, THL30 exhibited exceptional cyclic stability, with minimal hysteresis, negligible residual strain, and nearly overlapping stress‐strain curves (Figure S14). Furthermore, the mechanical loss of THL30 (tan δ = 0.41 at 1 Hz, 25°C) was significantly lower than that of the commercial VHB 4910 (tan δ = 0.93 at 1 Hz, 25°C), further confirming its advantage in dynamic response (Figure S15).

The dielectric constant (*ε*') and dielectric loss (tan *δ*_e_) of THL elastomers were evaluated. Benefiting from the strongly polar C─F bonds and the interfacial polarization, THL30 exhibited a high dielectric constant of 12.5 at 1 kHz. However, at higher HDFDA contents (≥50 mol%), the highly ordered rigid smectic phases significantly restricted the orientational polarization capability of the dipoles, thereby leading to a decrease in dielectric constant (Figure 2h). Furthermore, as the degree of phase separation intensified, the interfacial area between the two phases increased significantly, thereby enhancing the interfacial polarization relaxation loss [42, 43]. The tan *δ*_e_ increased from 0.013 for THL30 to 0.027 for THL100 (Figure S16), which compromised the energy conversion efficiency in practical applications. The electrical breakdown strength (*E*_b_) of the DEs was evaluated using a two‑parameter Weibull distribution function. As the HDFDA content increased, *E*_b_ significantly improved from 20.22 to 42.50 V µm^−1^ (Figure 2i). Mechanically, the rigid domains restricted chain mobility and raised the Young's modulus, which improved resistance to Maxwell stress and suppressed breakdown propagation [44]. Electrically, thermally stimulated depolarization current (TSDC) analysis revealed that the interfacial polarization relaxation peak shifted to higher temperatures with increasing HDFDA content, which indicated deeper trap energy levels at the phase interfaces (Figure 2j) [28]. Meanwhile, an additional deep‑trap peak from the smectic phase emerged. These deep traps effectively captured electrons, thus enhancing the *E*_b_ of the DEs.

### 2.3 Self‐Healing and Hierarchical Mechanical Response

Based on the above composition‐structure‐property evolution, THL30 exhibited optimized overall performance. We systematically investigated the self‐healing and strain‐stiffening behaviors of THL30. As quantitatively presented in Figure 3a, after 12 h of healing at room temperature, the calculated healing efficiency based on the recovered elongation reached as high as 95%, and that based on toughness attained 86%. In addition, the restored functionality is notable: the *E*_b_ (Figure S17) and the actuation area strain (Figures S18 and S19) retained about 83% and 71% of their initial values, respectively (Figure 3b). This discrepancy is mainly attributed to the different sensitivities of these properties to defects. Notably, healed THL30 still achieved an actuation area strain exceeding 150% at 45 V µm^−1^ and exhibited stable cycling over 5,000 cycles (Figure S20). A more intuitive demonstration was provided in Figure 3c, where an unstained sample and a sample stained with safranine T dye could heal together within 30 min, and be stretched to 5–8 times its original length. Such excellent self‐healing ability primarily originated from the rapid interchain diffusion and the reestablishment of physical interactions [45], as was revealed by a discernible diffusion interlayer at the interface of the two healed pieces.

**FIGURE 3:**
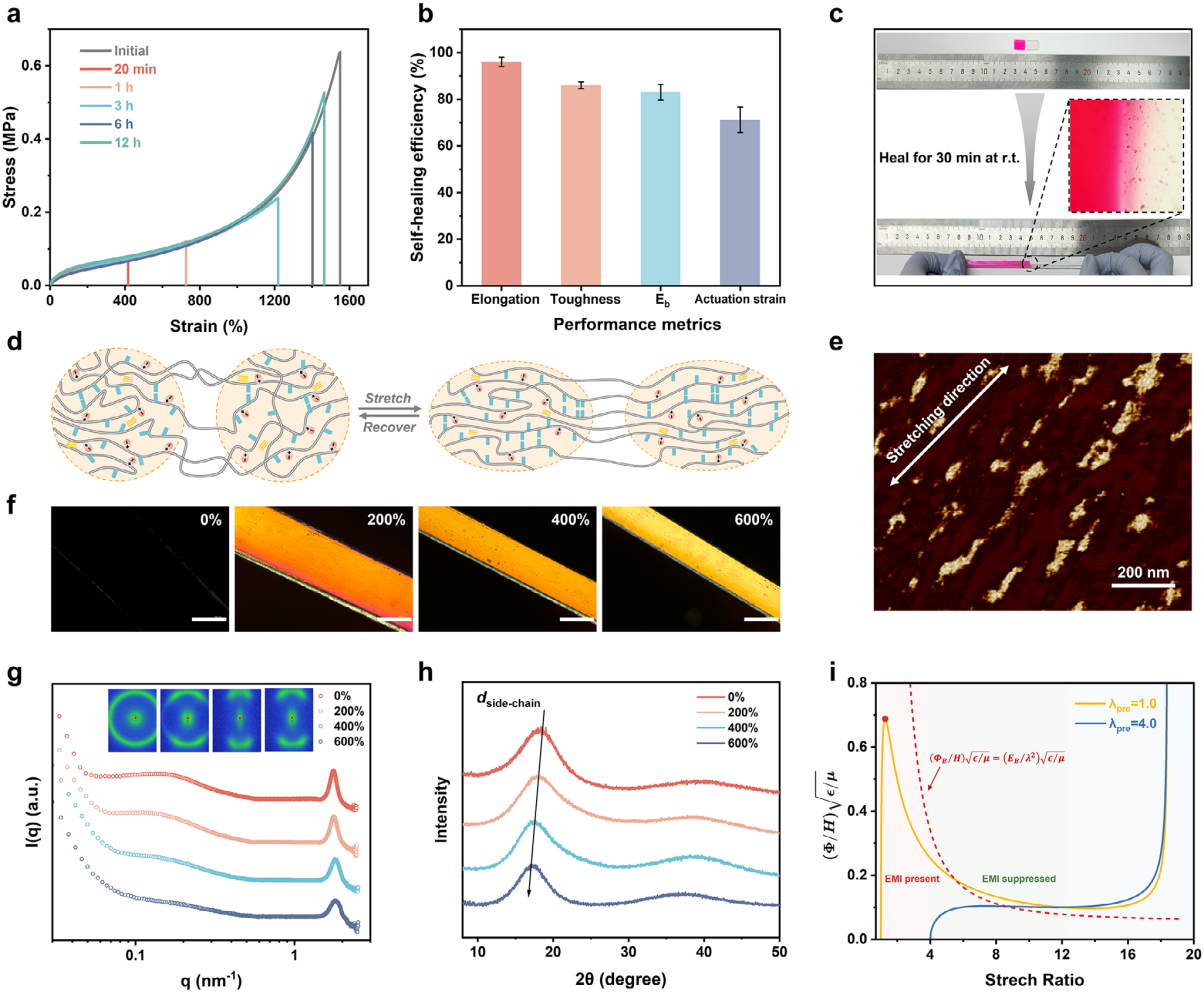
Self‐healing capability and strain‐dependent microstructure of THL30. (a) Tensile curves of THL30 healed for different periods at room temperature. (b) Self‐healing efficiency of the THL30 based on different performance metrics. (c) Two pieces of healed elastomer can be stretched as a whole after healing at room temperature for 30 min. Inset: Microscopic observation of the diffusion interlayer between two DEs healed at room temperature for 48 h. A diffusion region with tightly adhered and interlocked interfaces was clearly observed, suggesting an interchain‐diffusion‐mediated self‐healing mechanism. (d) Schematic diagram of the cooperative orientation of hard domains in THL30 under stress to resist electromechanical instability. (e) AFM phase images of THL30 stretched to 400% of its original length. (f) POM images of THL30 under different tensile strains. (g) 1D SAXS of THL30 under different tensile strains. Inset: Corresponding 2D SAXS patterns. (h) XRD profiles of THL30 under different tensile strains. (i) Voltage‐stretch response of DE membrane at λ_pre_ = 1.0 and λ_pre_ = 4.0, with electromechanical instability denoted with solid red dots.

Besides, the dynamic network provided a hierarchical mechanical response that effectively prevented catastrophic electrical breakdown from excessive thinning under high electric fields (Figure 3d). In the undeformed state, THL30 exhibited a typical isotropic phase‐separated structure, in which the HDFDA‐enrich domains were uniformly dispersed within the soft phase (Figure 2a). Upon pre‐stretch, the soft‐phase chain segments extended while the HDFDA hard domains rearranged and underwent significant elongated deformation (Figure 3e). In situ POM directly revealed a highly homogeneous structural orientation of THL30 along the stretching direction (Figure 3f). To further probe the origin of this orientation, in situ small‐angle x‐ray scattering (SAXS) was employed. The one‐dimensional scattering profiles in Figure 3g exhibited two key features: the peak at *q* = 1.76 nm^−1^ reflected the interlayer distance of the perfluoroalkyl side chains within the HDFDA rigid domains [39], while the peak at *q* = 0.11 nm^−1^ corresponded to the phase‑separated structures with a characteristic size of approximately 60 nm. As the strain increased, the high‑*q* peak shifted to the right, indicating a reduced interlayer distance and tighter layer packing. Meanwhile, the two‑dimensional SAXS patterns evolved from isotropic rings to anisotropic ellipses, implying alignment and elongation of the hard domains. The molecular‐scale structural evolution was elucidated by in situ XRD. Upon stretching, the initially disordered perfluoroalkyl side chains adopted a more perpendicular orientation relative to the main chains, causing the characteristic diffraction peak to gradually shift to lower 2θ angles (Figure 3h). This shift was attributed to an increased degree of ordering of the fluorinated pendant groups. This strain‐induced orientation manifests as a pronounced strain‐stiffening effect, which effectively suppresses EMI. According to the model proposed by Suo [46], the gradual alignment of rigid domains under pre‐stretch completely eliminates the peak in the voltage‐stretch curve (Figure 3i), a feature essential for excellent actuation performance.

### 2.4 Actuation Properties

Actuation sensitivity (*β*), defined as the ratio of the dielectric constant to Young's modulus, serves as a key metric to evaluate the theoretical actuation performance of DEs. As shown in Figure 4a, benefiting from the synergy between low Young's modulus and high dielectric constant, THL30 exhibited the highest actuation sensitivity among all samples, with a *β* value of 156.25, approximately 7.22 times that of VHB 4910. This significantly surpassed most recently reported high‐performance DEs [12, 23, 47, 48, 49, 50]. To evaluate the actual actuation performance, static actuation tests were conducted on THL0, THL10, THL30, and THL50. Without pre‐stretch, THL30 achieved a maximum areal strain of 32% at 20 V µm^−1^, significantly outperforming the other samples (Figure S21). After 300% pre‐stretch, the actuation strain of THL30 reached 235% at 52 V µm^−1^ (Figure 4b and Movie S1). As shown in Figure S22, owing to the optimized actuation sensitivity and film fabrication process, all DEs can be effectively actuated at voltages below 1 kV. A 20‐µm‐thick THL30 film showed maximum area strains of 38%, 72%, and 125% under applied voltages of 500 V, 700 V, and 900 V, respectively (Figure 4c).

**FIGURE 4:**
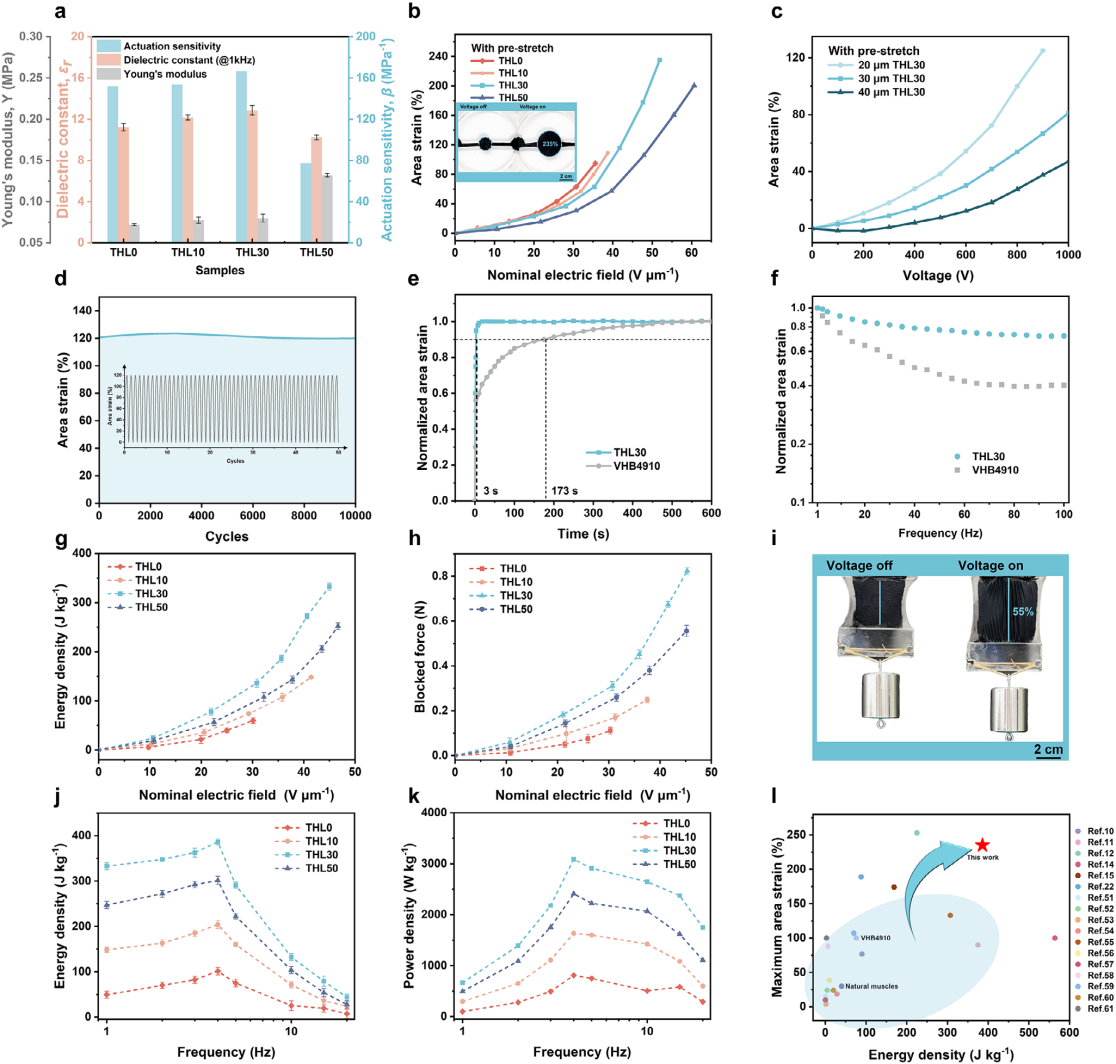
Actuation properties, energy density, and power density of THL elastomers. (a) Comparison of dielectric constant measured at 1 kHz, Young's modulus, and actuation sensitivity among THL elastomers. Error bars show s.d., *n* = 3. (b) The actuation area strains of THL elastomers under various electric fields with 300% biaxial pre‐stretch. Inset: photographs of the actuation area strain of THL30 at 52 V µm^−1^. (c) The actuation area strains of ultra‐thin THL30 films as a function of voltage. (d) Cyclic actuation of THL30 at 40 V µm^−1^ and 1 Hz for 10,000 cycles with 300% biaxial pre‐stretch. Inset: The area strain in the initial 50 cycles. (e) Time‐dependent response of VHB 4910 and THL30 at 40 V µm^−1^ with 300% biaxial pre‐stretch. The actuation area strain at 600 s was normalized to 1. (f) Frequency response of VHB 4910 and THL30 at 40 V µm^−1^ with 300% biaxial pre‐stretch from 1 to 100 Hz. The actuation area strain at 1 Hz was normalized to 1. (g) Energy density of THL elastomers at different electric fields with a 200 g load. Error bars show s.d., *n* = 3. (h) Blocked force of THL elastomers at different electric fields with 1 N pre‐load. Error bars show s.d., *n* = 3. (i) Photographs of linear actuation of THL30 pure‐shear DEA at 45 V µm^−1^ with a 200 g load. (j) Energy density of THL elastomers at different frequencies. The driving electric field was fixed at 30 V µm^−1^ for THL0, 40 V µm^−1^ for THL10, 45 V µm^−1^ for both THL30 and THL50. (k) Average power density of THL elastomers at different frequencies. (l) Ashby plot summarizing maximum area strain to energy density of this work and other DEs. VHB 4910 represented the measured value in this work.

Additionally, THL30 exhibited outstanding cyclic stability, rapid strain response, and excellent frequency adaptability. As illustrated in Figure 4d, under a driving frequency of 1 Hz and a large strain of 120%, THL30 operated stably for 10,000 cycles with no significant decay. Moreover, THL30 demonstrated an exceptionally fast time response. It reached 90% of its final strain within just 3 s and remained stable thereafter, significantly outperforming the VHB 4910, which required at least 170 s to achieve the same (Figure 4e). Regarding frequency response under an electric field of 40 V µm^−1^, as the driving frequency increased from 1 to 100 Hz, the actuation area strain of VHB 4910 dropped sharply to 50% of its maximum value, indicating pronounced viscoelasticity at high frequencies. Conversely, THL30 retained 72% of its maximum actuation area strain even at 100 Hz, demonstrating superior frequency adaptability (Figure 4f). Notably, THL0, THL10, and THL50 also outperformed VHB 4910 in dynamic response (Figure S23).

Energy density and power density are determining factors for DEs in actuation and energy harvesting applications. Here, these two quantities were characterized for a single‐layer THL30 under pure shear mode. Specifically, the DE film was uniaxially pre‐stretched and then fixed onto a rigid frame to maintain the pre‐strain. Upon voltage application, the DEA expanded freely along the loading direction, generating linear actuation and converting electrical energy into mechanical work. Figure 4g compared the energy densities of THL0, THL10, THL30, and THL50 under a load of 200 g. The results indicated that THL30 achieved an energy density of 332 J kg^−1^ at 45 V µm^−1^ (Movie S2), producing a blocked force of 0.82 N (Figure 4h), which corresponded to a stress of approximately 0.39 MPa and a linear strain of 55% (Figure 4i). This energy density was substantially higher than that of the other THL samples. Frequency dependence of the energy and power densities of THL30 was further characterized. At a frequency of 4 Hz, THL30 attained a maximum energy density of 386 J kg^−1^ (Figure 4j), approximately 9.65 times that of natural muscle (40 J kg^−1^) [12, 22, 51], with a specific power of 3089 W kg^−1^ (Figure 4k). Nevertheless, it remained at a relatively high level of 132 J kg^−1^ at 10 Hz, corresponding to a power density of 2647 W kg^−1^. THL30 demonstrates the most exceptional combination of actuation strain and energy density among all DEs reported in recent years (Figure 4l) [10, 11, 12, 14, 15, 22, 51, 52, 53, 54, 55, 56, 57, 58, 59, 60, 61]. Notably, THL30 maintained stable performance during prolonged operation. During a 10 000‐cycle test conducted at 40 V µm^−1^ and 4 Hz (Figure S24), THL30 consistently delivered an energy density of 280 J kg^−1^ and a power density of 2240 W kg^−1^ under a 200 g load, strongly validating its potential as a next‐generation high‐performance artificial muscle.

### 2.5 Biomimetic Swimming Robot Actuation

Inspired by the locomotion mechanism of purely aquatic frogs, we fabricated a biomimetic swimming robot based on THL30. As drag‐based paddlers, purely aquatic frogs derive propulsion primarily from the kicking motion of their webbed feet, while their forelimbs are used for support and steering [62]. Figure 5a shows that a complete swimming cycle can be divided into three phases: (a) the propulsion phase, (b) the gliding phase, and (c) the recovery phase.

**FIGURE 5:**
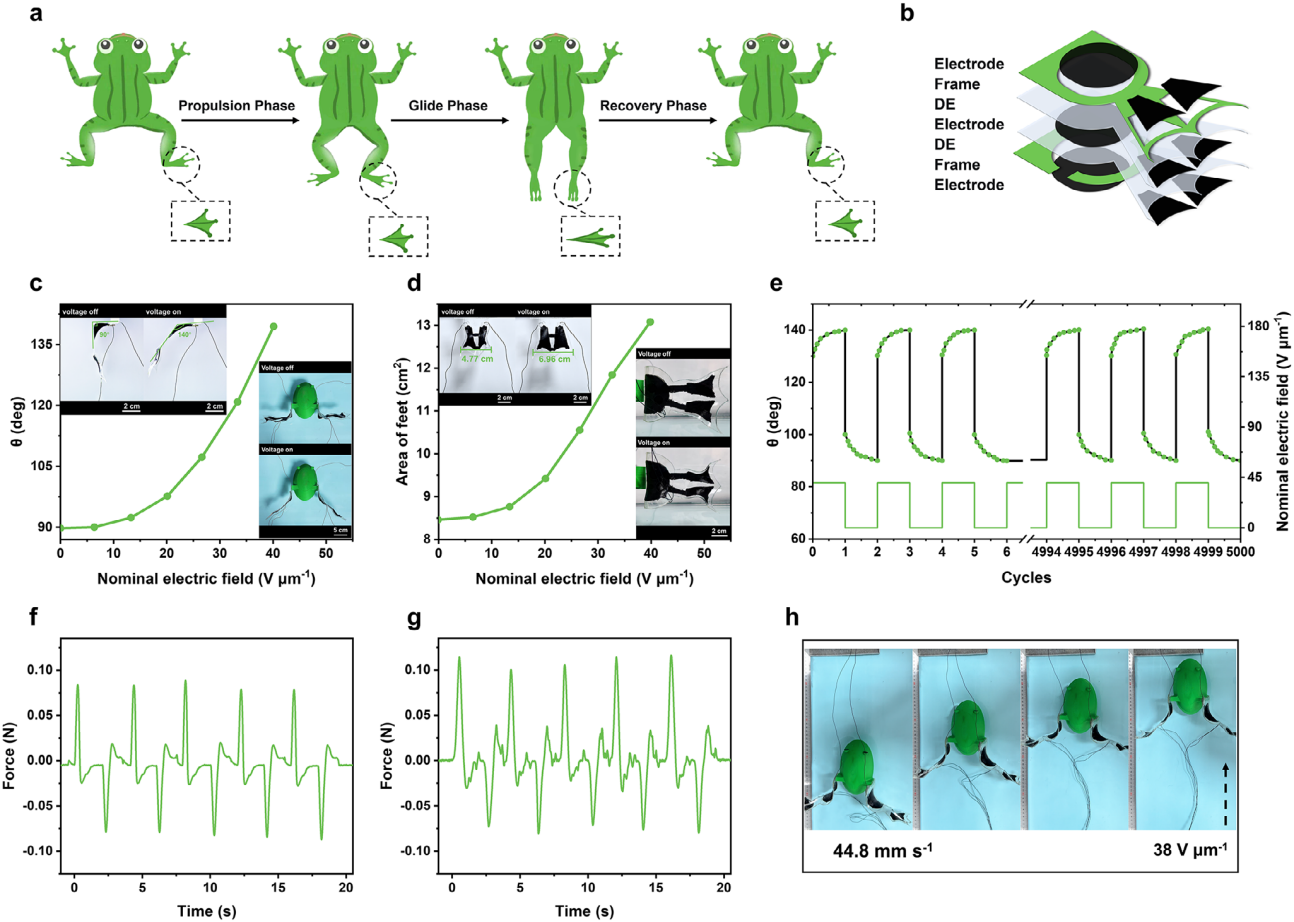
Biomimetic swimming robot based on THL30. (a) Schematic illustration of the swimming locomotion of frogs. (b) Schematic illustration of the layer structure of the biomimetic swimming robot. (c) Bending angle of the leg actuator as a function of electric field. Inset: photographs of the actuated leg actuator. (d) Projected area of the foot actuator as a function of electric field. Inset: photographs of the actuated foot actuator. (e) Cyclic stability of the bending angle over 5000 actuation cycles. (f) Thrust force with leg‐only actuators under square‐wave activation. (g) Thrust force with both the leg and the foot actuators under square‐wave activation. (h) Photographs of the THL30‐based biomimetic swimming robot swimming at 38 V µm^−^^1^ and 1 Hz.

Informed by the aforementioned biological prototype, a swimming robot driven by THL30 was designed. The robot body was fabricated using three‐dimensionally printed polylactic acid (PLA). The actuator consisted of two units: one mimicking the hind limb of the frog and the other mimicking the webbed foot. The DEA adopted a multilayer stacked structure, formed by alternating three layers of compliant electrodes with two layers of THL30 (Figure 5b). The actuation performances of the leg and foot actuators were evaluated separately. For the leg actuator, as illustrated in Figure 5c, the bending angle gradually increased from 90° to 140° with the increasing driving voltage (Movie S3). For the foot actuator, the projected area was used as the quantitative metric for actuation capability (Figure 5d). The results showed that at 40 V µm^−1^, the projected area of the foot increased by over 60% compared to its initial state (Movie S4). These results suggested that both the leg and foot actuators effectively mimic the typical swimming motions of a frog. In addition, as shown in Figure 5e, after 5,000 cycles, the bending angle of the leg actuator exhibited only a minor deviation of 2°, demonstrating excellent cyclic stability. Moreover, towing tests were conducted using the actuator. Under a driving voltage of 40 V µm^−1^, the actuator successfully towed wooden blocks weighing 3 g, 6 g, and 9 g over distances of 4.5 cm, 3.5 cm, and 3 cm, respectively (Figure S25). This highlighted the capability of the THL30‐based actuator to deliver mechanical output under external loading conditions.

Notably, the high electronegativity of fluorine and the strong electrostatic characteristics of C─F bonds endow THL30 with high hydrophobicity, as indicated by a water contact angle of 118° (Figure S26). After immersion in deionized (DI) water and simulated seawater (3.5 wt.% NaCl) at room temperature for 7 days, THL30 maintained stable mechanical properties (Figures S27 and S28) and dielectric performance (Figures S29 and S30), with no visible swelling, dissolution, cracking, or delamination. To further evaluate underwater operational stability, the THL30‐based actuator with more water‐stable SWCNT electrodes (Figure S31) was cycled at 35 V µm^−1^ and 0.5 Hz. Its bending angle was approximately 60° initially and remained at approximately 58° after 5 000 underwater cycles (Figure S32).

The thrust force generated by the THL30‐based actuators was measured in water. The two actuators were mounted on an inverted U‐shaped acrylic frame and placed in water. The U‐shaped frame was connected to a force sensor via an acrylic rod to measure the thrust force generated by the actuators in the Z‐direction (Figure S33). Figure 5f presented the time‐dependent thrust force measured by the force sensor under a square‐wave voltage of 40 V µm^−1^. Positive and negative values represented thrust force and drag force, respectively. When only the leg actuator was activated, the average peak thrust force and average peak drag force were 0.085 N and 0.072 N, respectively. In contrast, when both the leg and foot actuators were activated simultaneously, the average peak thrust force significantly increased to 0.120 N, while the average peak drag force remained at 0.073 N (Figure 5g). The synergistic contribution of the foot actuator resulted in a 41.2% increase in thrust force. The results clearly demonstrated that the voltage‐induced expansion of the foot actuator effectively enlarged the water‐pushing area, thereby ensuring thrust force substantially exceeded drag force throughout a swimming cycle. As shown in Movie S5, the biomimetic swimming robot achieved stable forward locomotion in water with an average speed of 44.8 mm/s (Figure 5h). Notably, the total mass of the two actuators was only 8.2 g, accounting for approximately 9.4% of the total robot mass (87 g), highlighting the significant advantages of lightweight design. THL30 shows considerable promise for underwater actuation, but its practical deployment is constrained by factors such as extreme water temperatures, long‐term thermal aging, and biofouling. Although its hydrophobic surface can mitigate attachment, long‐term antifouling and encapsulation strategies still warrant further investigation.

## 3 Conclusion

In summary, we propose a phase‐separated fluorinated polyacrylate DE in which HDFDA spontaneously assembles into rigid domains via fluorine‐fluorine interactions. Upon stretching, these domains undergo cooperative orientation, establishing a titin‐like hierarchical strain‐stiffening effect that effectively suppresses EMI. Meanwhile, the synergy among multiple dynamic physical interactions endows the DE with rapid responsiveness and self‐healing capability. This strategy enables the DE with a high dielectric constant and mechanical flexibility to simultaneously achieve large actuation strains and ultrahigh energy density under low electric fields. As a demonstration, a biomimetic swimming robot constructed from this DE achieves efficient aquatic locomotion. Overall, this work provides a novel strategy for developing high‐performance DEs, thereby paving the way for the advancement of soft robotics.

## Author Contributions

**Chengzhen Du**: writing – original draft, conceptualization, methodology, investigation, visualization, writing – review and editing. **Lijuan Yin**: conceptualization, methodology, writing – review and editing. **Yujie Chen**: conceptualization, methodology, supervision, funding acquisition, writing – review and editing, writing – original draft. **Xichen Yang**: conceptualization, investigation, methodology, validation. **Zhi‐Min Dang**: conceptualization, methodology, writing – review and editing, supervision. **Zimo Pang**: conceptualization, investigation, methodology, validation. **Sijia Li**: conceptualization, investigation, methodology, validation. **Qunfu Fan**: conceptualization, methodology. **Hezhou Liu**: conceptualization, methodology, writing – review and editing, supervision, funding acquisition.

## Conflicts of Interest

The authors declare no conflicts of interest.

## Supporting information




**Supporting File 1**: advs78715‐sup‐0001‐SuppMat.docx.

**Supporting File 2**: advs78715‐sup‐0002‐MovieS1.mov.

**Supporting File 3**: advs78715‐sup‐0003‐MovieS2.mov.

**Supporting File 4**: advs78715‐sup‐0004‐MovieS3.mov.

**Supporting File 5**: advs78715‐sup‐0005‐MovieS4.mov.

**Supporting File 6**: advs78715‐sup‐0006‐MovieS5.mov.

## Data Availability

The data that support the findings of this study are available from the corresponding author upon reasonable request.
