# Supplementary material for: Titin‐Mimetic Phase Separation Enables a High‐Performance Dielectric Elastomer for Artificial Muscles
Source: Adv Sci (Weinh). 2026 Nov 9:e78715. Online ahead of print. doi: 10.1002/advs.78715 (PMC13748901; doi:10.1002/advs.78715)
Supplement: Supplementary file 1 [file ADVS-9999-e78715-s005.docx]

**Supplementary Information**

**Titin-Mimetic Phase Separation Enables a High-Performance Dielectric Elastomer for Artificial Muscles**

*Chengzhen Du,^1^* *Lijuan Yin,^2^ Yujie Chen,^1, 4,^* *^*^ Xichen Yang,^1^ Zhi-Min Dang,^3, *^ Zimo Pang,^1^ Sijia Li,^1^ Qunfu Fan,^1^ and Hezhou Liu^1, 4, *^*

*^1^* School of Materials Science and Engineering, State Key Laboratory of Metal Matrix Composites, Shanghai Jiao Tong University, Shanghai 200240, China.

*^2^* State Key Laboratory of Smart Power Distribution Equipment and System, Hebei University of Technology, Tianjin 300387, China

*^3^* State Key Laboratory of Power System Operation and Control, Department of Electrical Engineering, Tsinghua University, Beijing 100084, China

*^4^* National Engineering Research Center of Special Equipment and Power System for Ship and Marine Engineering, Shanghai 200030, China

* Corresponding authors: Yujie Chen, Zhi-Min Dang, Hezhou Liu

E-mail addresses: [yujiechen@sjtu.edu.cn](mailto:yujiechen@sjtu.edu.cn); [dangzm@tsinghua.edu.cn](mailto:dangzm@tsinghua.edu.cn); [hzhliu@sjtu.edu.cn](mailto:hzhliu@sjtu.edu.cn).

**Experimental**

**Materials**

2,2,2-Trifluoroethyl acrylate (TFEA) and 1H,1H,2H,2H-Heptadecafluorodecyl acrylate (HDFDA) were purchased from Shanghai Macklin Biochemical Co., Ltd. Lauryl acrylate (LA) was purchased from J&K Scientific Ltd. 2-Hydroxy-2-methylpropiophenone (HMPP), as a photo-initiator, was purchased from Shanghai Macklin Biochemical Co., Ltd. Carbon conductive grease (#846) was purchased from MG Chemicals. The aqueous dispersion of ultrapure single-walled carbon nanotubes (SWCNTs) (0.15 wt%) was obtained from Nanjing XFNANO Materials Tech Co., Ltd. The VHB 4910 used for comparison with the DE was purchased from 3M Company. All the chemicals were used as received without further purification.

**Preparation of DE films**

For the preparation of DE films, each component was weighed in the proportions shown in Table S1. For THL30, 7 mmol TFEA, 3 mmol HDFDA, 1 mmol LA and 0.05 mmol HMPP were mixed and stirred overnight to obtain a well-mixed precursor solution. To ensure an oxygen-free environment during the photopolymerization process, a custom-made container was designed and fabricated. The container consisted of two glass plates with a layer of polytetrafluoroethylene sandwiched as a spacer, effectively isolating the air and ensuring a smooth curing process. Next, the precursor solution was slowly injected into the mold and subjected to vacuum deaeration for 10 minutes. Subsequently, it was polymerized under a UV lamp (365 nm) for 4 hours. To prepare other THL samples with different HDFDA contents, only the fractions of TFEA and HDFDA were tuned while the concentration of LA was fixed to 1 mmol.

**Mechanical properties characterization**

Mechanical performance tests were conducted using a microcomputer-controlled electronic universal testing machine (LD23, Shenzhen) at room temperature. The samples were dumbbell-shaped with a parallel part measuring 10 mm (length) × 4 mm (width) × 0.5 mm (thickness). The tensile rate was 50 mm min^-1^. All mechanical tests were conducted three replicate specimens to ensure reproducibility. The stress to strain ratio at 5% strain was defined as the Young's modulus of the sample based on the obtained stress-strain curve. A cyclic tensile test was performed at 400% strain, with 10 cycles of loading and unloading at a rate of 50 mm min^-1^. Self-healing tests were performed at room temperature. The samples were cut using a sharp knife and then the cut pieces were gently recontacted in air and left to heal for the specified period. Mechanical tests were conducted after the designated healing time at a loading rate of 50 mm min^-1^. Each mechanical test was repeated with at least three individual samples.

**Dielectric properties characterization**

The dielectric properties were measured with the E4990A Impedance Analyzer at room temperature. The samples were cut into circular discs with a diameter of 10 mm and a thickness of 0.5 mm, and the frequency range of the measurements was 10^2^ Hz to 10^6^ Hz. The electrical breakdown strength of the dielectric elastomer was measured using a Huace HCDJC series 100 kV breakdown voltage tester, following the GB/T 1408.1-2016 standard. The samples were fixed between parallel electrodes with dimensions of *φ*25 mm and *φ*75 mm, immersed in silicone oil at room temperature, and subjected to a DC voltage ramp of 500 V s^-1^. For each formulation, ten samples were tested, and the breakdown event was identified as the voltage value when the current through the sample reached 20 mA. The electrical breakdown strength (*E*_b_) was characterized using a two-parameter Weibull distribution function, $P=1-e^{(-{(E/E_{b})}^{\gamma})}$ , where *P* is the cumulative probability of electrical failure across 10 specimens, *E* is the measured electrical breakdown strength for each sample, *E*_b_ is the breakdown strength corresponding to 63.2% of the sample failures, and *γ* is the slope parameter that assesses the data dispersion.

**Other characterizations**

The liquid-state ^1^H NMR, ^13^C NMR, and ^19^F NMR spectra were measured on a Bruker Avance III 600 MHz NMR spectrometer. Gel permeation chromatography (GPC) measurement was performed by Tosoh HLC-8420GPC using dimethyl formamide (DMF) as eluent. FTIR spectra were recorded using a Spectrum 100 FT-IR spectrometer in the range of 500-4000 cm^-1^. The transparency of dielectric elastomer films (thickness ~500 μm) was evaluated using a UV-visible spectrophotometer (Lambda 950, PerkinElmer). Polarized optical microscopy (POM) images were taken on a polarizing microscope (DM4P, Leica). Differential scanning calorimetry (DSC) was performed using TA Instruments DSC 250 from -50 °C to 150 °C with a heating rate of 5 °C min^-1^. X-ray diffraction (XRD) patterns were collected on a Cu target X-ray diffractometer (D8 ADVANCE Da Vinci, Bruker). The diffraction angle ranged from 3° to 60°. Atomic Force Microscope (Dimension XR, Bruker) was used to observe the microphase separation structure of the dielectric elastomers. Dynamic mechanical analysis (DMA) was performed on a TA Q850 dynamic mechanical analyzer. The temperature was increased at a rate of 5 °C per minute, with a frequency of 1 Hz. The temperature range was from -40 °C to 100 °C. Low-field ^19^F NMR tests were carried out on a VTMR20-010V-I NMR analyzer (Niumag). The ^19^F NMR probe was used to measure the *T*_2_ values of samples. SAXS measurement of dielectric elastomer was carried out with a Xeuss 3.0 system (Xenocs SA). For strain-dependent AFM observations, the dielectric elastomer film was first stretched to a specific strain and then attached to a glass slide. Thermally stimulated depolarization current (TSDC) was recorded using a TSC6520 thermally stimulated current measurement system. The sample was polarized at 80°C for 30 min, and then rapidly cooled to -100°C and kept for 10 min. Finally, the sample was heated to 150°C at a rate of 3°C/min, and the current was recorded. Contact angles were measured using a KRÜSS DSA100 contact angle goniometer (KRÜSS, Germany) with deionized water as the testing liquid. At least three positions were measured for each sample.

**Density Functional Theory calculations**

In this work, geometry optimizations were carried out with density functional theory (DFT) as implemented in Gaussian 16, using the B3LYP functional (with GD3BJ dispersion correction) and the 6-311G* basis set. All identified stationary points were confirmed to be stable by the absence of imaginary frequencies in their vibrational modes. The prediction of interaction sites was conducted based on the molecular electrostatic potential visualized using Gauss View.

**Molecular dynamics simulation**

Using the GROMACS 2020.6 software with a custom force field, molecular dynamics simulations were performed on the THL30 system. The system was first subjected to thorough energy minimization under the custom force field to eliminate unfavorable contacts, followed by NVT (canonical ensemble) equilibration to stabilize the temperature, and then switched to NPT (isothermal-isobaric ensemble) production simulation to obtain an equilibrium trajectory. The final analysis was conducted using the production phase trajectory. Throughout the simulation, the equations of Newtonian motion were integrated using a standard molecular dynamics time step. Temperature and pressure were maintained using standard thermostat and barostat algorithms, respectively. Electrostatic interactions were treated with the particle mesh Ewald (PME) method, with real-space and reciprocal-space cutoff parameters set according to conventional practices. The cutoff distance for van der Waals interactions was set to the standard value. For trajectory analysis, the built-in analysis tools of GROMACS were used to extract fluorine atom coordinates from the entire trajectory, and the F–F radial distribution functions for HDFDA-HDFDA, HDFDA-TFEA, and TFEA-TFEA were calculated separately.

**Modeling studies on electromechanical behaviors of DEs**

We used Suo’s model to explain the electromechanical behaviors of our elastomers. For a dielectric elastomer membrane under equal‑biaxial stretching, the mechanical equilibrium equation is:

$$\sigma+\varepsilon E^{2}=f\left( \lambda\right)$$

where $\sigma=\frac{P}{\lambda h}$ is the applied mechanical stress arising from the preload, $E=\frac{\Phi}{h}$ is the true electric field, $h=\frac{H}{\lambda^{2}}$ is the instantaneous thickness under incompressibility, and $f\left( \lambda\right)$ represents the elastic stress–stretch relation of the material. Substituting these definitions yields the explicit voltage-stretch relationship:

$$\Phi=\frac{H}{\sqrt{\varepsilon}}\lambda^{-2}\sqrt{f\left( \lambda\right)-\frac{P\lambda}{H}}$$

Here, $P$is the preload per unit width, $\lambda$ is the equi‑biaxial stretch ratio, $H$ is the initial film thickness, and $\Phi$ is the applied voltage. The dielectric breakdown voltage is similarly expressed as:

$$\Phi_{B}=E_{B}H\lambda^{-2}$$

Where $\Phi_{B}$is the breakdown voltage and $E_{B}$is the dielectric breakdown strength.

For hyperelastic materials under a constant equal‑biaxial prestress, the equilibrium equation is further expressed in the form:

$$\frac{P}{\mu H}+\frac{\varepsilon}{\mu}{(\frac{\Phi}{H})}^{2}\lambda^{3}=\zeta\sqrt{n}\frac{{\lambda-\lambda}^{-5}}{3\Lambda}$$

where $\mu$is the small‑strain shear modulus, $\zeta$ is the normalized force in each polymer chain, and $n$ is the number of statistical links per chain, which governs the chain flexibility and the limiting stretch $\lambda_{lim}$. The average chain stretch $\Lambda$satisfies the Langevin relation:

$$\Lambda=\sqrt{n}(\frac{1}{\tan h\zeta}-\frac{1}{\zeta})$$

and the limiting stretch is related to $n$ by:

$$n=\frac{2\lambda_{lim}^{2}+\lambda_{lim}^{-4}}{3}$$

with the condition $\Lambda=\sqrt{n}$ at the fully stretched state. This set of equations provides a quantitative basis for analyzing the coupled electromechanical response and for evaluating the onset of electromechanical instability in our materials.

**Static actuation performance measurement**

The actuation strain of the dielectric elastomers was quantified by measuring the change in area before and after actuation. The nominal electric field was calculated by dividing the applied voltage by the initial thickness of the electroactive elastomer film before actuation.

For DEAs without pre-stretch, the sample was fixed onto a PET annular frame with an inner diameter of 20 mm (*d* = 20 mm). Carbon grease was coated on both sides of the film to serve as compliant electrodes. The actuation area was circular with a diameter of 1 cm. The high-voltage power supply (AMR10R20, Wisman) was gradually increased from 0 V until electrical breakdown occurred in the DEAs. During this process, a laser sensor (ILD1420-10, Micro-Epsilon) measured and recorded the out-of-plane displacement (*h*) of the actuation center, and the final area strain was determined using equation $S={(2h/d)}^{2}\times100\%$.

For the pre-stretched DEA, all samples were biaxially pre-stretched by 300% and fixed onto a PET annular frame with an inner diameter of 80 mm. Similarly, carbon grease was coated on both sides of the film to serve as compliant electrodes. The actuation area was circular with a diameter of 1 cm. During the actuation process, the electrically active area expanded and the thickness decreased, which was captured by a camera. The high-voltage power supply was gradually increased from 0 V until electrical breakdown occurred in the DEAs. Afterwards, the actuation strain was analyzed using ImageJ, and the final area strain was determined using equation $\frac{a_{1-}a_{0}}{a_{0}}\times100\%$, where *a*_1_ is the actuated area under high voltage, and *a*_0_ is the initial area.

The DE film was cut using a sharp knife and then the cut pieces were gently re-contacted and healed in air for a certain time. For DEAs without pre-stretch, the healed DE film was fixed onto a PET annular frame with an inner diameter of 20 mm (*d* = 20 mm). Carbon grease was coated on both sides of the film to serve as compliant electrodes. For DEAs with pre-stretch, the healed DE film pre- was stretched by 300% and fixed onto a PET annular frame with an inner diameter of 80 mm. Similarly, carbon grease was coated on both sides of the film to serve as compliant electrodes. The high-voltage power supply was gradually increased from 0 V until electrical breakdown occurred in the DEAs.

**Dynamic actuation performance measurement**

Dynamic actuation performance was evaluated using DEAs with pre-stretch. The actuation area was circular with a diameter of 1 cm. For the time response, the nominal electric field applied on the elastic film was 40 V μm^-1^, and the DEA was driven continuously for 600 s. For the frequency response, the nominal electric field applied on the elastic film was 40 V μm^-1^, and the frequency was gradually increased from 1 Hz to 100 Hz to analyze the frequency response characteristics of DEAs. For the cycle tests, the DEA was subjected to a nominal electric field of 40 V μm^-1^ at a frequency of 1 Hz for a total of 10000 actuation cycles.

**Energy density and power density measurements of DE films**

After biaxially pre-stretching a single-layer DE film by 300%, PET was adhered to the top and bottom edges of the DE film to confine it. Energy density and power density tests were conducted by suspending weights at the bottom of the sample. Under a load of 200 g, the effective actuation area of the DE film was 3 cm×3.5 cm and the thickness of the elastomer was 85 μm. The voltage was gradually increased from 0 V, and the actuation strain of the DE film during the contraction process was recorded using a camera. The energy density (*E*) of the DE film at different electric fields was determined based on the increase in potential energy during the contraction process using the formula $E=\frac{m_{L}gh}{m_{a}}$, where *m*_L_ is the mass of load, *m*_a_ is the mass of active area, *h* is the increased height of the load. Subsequently, a fixed voltage was applied to the DE film at 45 V μm^-1^, and the energy density and power density of the DE film at different frequencies were measured by varying the electric field frequency from 1 Hz to 20 Hz. The power density (*P*) of the elastic film was determined according to $P=2E\times f$, where *f* is the frequency. The blocked force was measured in pure-shear mode. When measuring the blocked force, the samples were clamped on both ends and preloaded with 1 N under isometric conditions.

**Preparation of SWCNT electrode**

SWCNT ink (0.15 wt%) was ultrasonicated for 10 min and then vacuum filtered onto a PTFE membrane (50 mm diameter, 0.1 μm pore size). After drying at room temperature for 48 h, the SWCNT film was stamp-transferred onto the surface of THL30 to form a SWCNT electrode.

**Fabrication of soft robot**

The robot leg and foot actuators consisted of three electrode layers separated by two layers of dielectric elastomer. The fabrication process was as follows: Two THL30 membranes were pre-stretched by 300%, and fixed each between two annular acrylic frames to maintain their pre-stretch. The PET frames with a thickness of 0.3 mm were cut into specific shapes using the laser cutting system (MC-3020, Mintron). Patterned SWCNT was applied to one side of the top THL30 membrane as the high voltage electrode. A 0.3 mm thick PET frame was stacked on the other side of the top THL30 membrane, and a SWCNT electrode was applied within the actuation area as the first grounded electrode. A 0.3 mm thick PET frame, which served as a component to restrict the direction of actuation, was stacked on another side of the bottom pre-stretched THL30 membrane. Patterned SWCNT was then applied as the second grounded electrode. Two THL30 membranes were stacked together to encapsulate the high voltage electrode. Upon removing the acrylic frames and surrounding THL30, the leg and foot assembly bent into a buckled shape with a characteristic curvature.

**Figure S1.** FTIR spectra of THL30 and THL30 precursor. The THL30 precursor was a mixture of TFEA, HDFDA, and LA with a molar ratio of 70:30:10. The FTIR spectra of the precursor of THL30 before and after polymerization showed that the absorption peak corresponding to the ν(C=C) (from acrylate) at 1637 cm^-1^ vanished, indicating that the conversion of the comonomers was almost 100%.

**Figure S2.** ^1^H NMR (acetone-d6) spectrum of THL30.

**Figure S3.** ^13^C NMR (acetone-d6) spectrum of THL30.

**Figure S4.** ^19^F NMR (acetone-d6) spectrum of THL30.

**Figure S5.** GPC curve of THL30.

**Figure S6.** Stress-strain curves of PTFEA with different LA contents. Strain softening in PTFEA due to irreversible chain sliding, which cannot be recovered in the subsequent releasing process. In contrast, the long alkyl side chains of LA construct a physical crosslinked network via entanglements, endowing the DE with excellent elastic recovery capability. Excessive LA content led to a significant increase in modulus and a sharp decrease in elongation at break. Therefore, we chose to add LA with a mole fraction of 10%.

**Figure S7.** SAXS curves of TFEA-LA0 and TFEA-LA10. No obvious scattering peaks were observed, indicating that the elastomer remains structurally homogeneous at the nanoscale.

**Figure S8.** POM images of THL elastomers. No birefringence was observed in the samples with HDFDA contents below 50 mol%, suggesting an amorphous structure. In contrast, apparent birefringence was observed in the samples with higher HDFDA contents, due to the self-assembly of HDFDA into ordered domains.

**Figure S9.** Statistics of modulus and toughness of THL elastomers.

**Figure S10.** Stress-strain curves of THL0, THL10, THL30 and THL50.

**Figure S11**. Side-chain average distance changes of THL elastomers. The distance was calculated with Bragg's equation from the XRD data in Figure 2e.

**Figure S12.** *T*_2_ relaxation times of THL elastomers derived from low-field ^19^F NMR spectra.

**Figure S13.** FTIR and corresponding second derivative spectra of THL elastomers in the ν_as_ (CF_2_) region.

**Figure S14.** a) Cyclic stress-strain curves of THL elastomers. b) Cyclic stress-strain curves of THL30 under 400% strain for 10 cycles.

**Figure S15.** Mechanical loss factor of THL elastomers from -40 °C to 100 °C.

**Figure S16.** Dissipation factor of THL elastomers from 10^2^ Hz to 10^6^ Hz.

**Figure S17.** Electrical breakdown strength of THL30 before and after self-healing for 12 h.

**Figure S18.** Actuation area strain of THL30 before and after self-healing for 12 h without pre-stretch.

**Figure S19.** Actuation area strain of THL30 before and after self-healing for 12 h with pre-stretch.

**Figure S20.** Cyclic actuation of THL30 at 40 V μm^-1^ and 1 Hz for 5000 cycles after self-healing for 12 h with pre-stretch.

**Figure S21.** Actuation area strains of THL elastomers under different electric fields without pre-stretch.

**Figure S22.** The actuation area strains of 20-μm-thick THL elastomers as a function of voltage.

**Figure S23.** Cyclic actuation of a) THL0 at 30 V μm^-1^ and 1 Hz, b) THL10 at 35 V μm^-1^ and 1 Hz, c) THL50 at 50 V μm^-1^ and 1 Hz, for 10,000 cycles with 300 % biaxial pre-stretch. Inset: The area strain in the initial 50 cycles. Time-dependent response of d) THL0 at 30 V μm^-1^ and 1 Hz, e) THL10 at 35 V μm^-1^ and 1 Hz, f) THL50 at 50 V μm^-1^ and 1 Hz, with 300 % biaxial pre-stretch. The actuation area strain at 600 s was normalized to 1. Frequency response of g) THL0 at 30 V μm^-1^, h) THL10 at 35 V μm^-1^, i) THL50 at 50 V μm^-1^, with 300 % biaxial pre-stretch from 1 Hz to 100 Hz. The actuation area strain at 1 Hz was normalized to 1.

**Figure S24.** The cyclic stability of a) power density and b) energy density of THL30 in 10000 cycles actuation test with a 200 g load. The electric field was 40 V μm^-1^ and the frequency was 4 Hz.

**Figure S25.** Photos of the soft actuator based on THL30 towing different loads at 40 V μm^-1^.

**Figure S26.** Water contact angle of THL30.

**Figure S27.** Stress–strain curves of THL30 after immersion in DI water and saline for 7 days. Inset: Photographs of THL30 after immersion in DI water and saline for 7 days. Both immersed samples retained mechanical properties comparable to their initial values.

**Figure S28.** Cyclic stress-strain curves of THL30 after immersion in (a) DI water and (b) saline for 7 days. The immersed samples exhibited nearly overlapping loading-unloading curves over 10 cycles at 400% strain, with no apparent increase in residual strain.

**Figure S29.** Dielectric constants and dissipation factors of THL30 after immersion in DI water and saline for 7 days. THL30 showed slight increases in dielectric constant and dielectric loss due to water molecular polarization and interfacial polarization.

**Figure S30.** Weibull distribution of electrical breakdown strength (n = 10) for THL30 after immersion in DI water and saline for 7 days. In saline, ions further intensified interfacial polarization and conduction loss, leading to a greater decrease in breakdown strength, which remained at only 84% of the initial value.

**Figure S31.** Sheet resistance of SWCNT electrodes after immersion in DI water and saline for 7 days. No obvious leaching, migration, or detachment of the electrodes was observed. The sheet resistance increased from an initial value of 63.8 kΩ/sq to 70.8 kΩ/sq in DI water and 77.3 kΩ/sq in saline solution.

**Figure S32.** Photographs of the actuator after the first and 5000th actuation cycles under water.

**Figure S33.** Schematic illustration of the force measurement set-up.

**Table S1.** Formulations (molar ratio) and nomenclature of pre-polymer solutions and corresponding elastomer films.

| **Samples** | **TFEA** | **HDFDA** | **LA** | **HMPP** |
| --- | --- | --- | --- | --- |
| **TFEA-LA0** | 100 | 0 | 0 | 0.5 |
| **TFEA-LA5** | 100 | 0 | 5 | 0.5 |
| **TFEA-LA10** | 100 | 0 | 10 | 0.5 |
| **TFEA-LA15** | 100 | 0 | 15 | 0.5 |
| **THL0** | 100 | 0 | 10 | 0.5 |
| **THL10** | 90 | 10 | 10 | 0.5 |
| **THL30** | 70 | 30 | 10 | 0.5 |
| **THL50** | 50 | 50 | 10 | 0.5 |
| **THL70** | 30 | 70 | 10 | 0.5 |
| **THL100** | 0 | 100 | 10 | 0.5 |
